# Bacterial vector-borne plant diseases: global issues caused by three-way interactions

**DOI:** 10.1007/s44297-025-00049-0

**Published:** 2025-05-08

**Authors:** Yixuan Huang, Jianan Hao, Xiaotian Tang

**Affiliations:** 1 Zhejiang Key Laboratory of Biology and Ecological Regulation of Crop Pathogens and Insects, Ministry of Agriculture Key Lab of Molecular Biology of Crop Pathogens and Insect Pests, Institute of Insect Sciences, College of Agriculture and Biotechnology, Zhejiang University, Hangzhou, China; 2 College of Plant Protection, Hunan Agriculture University, Changsha, China; 3 Department of Entomology, Texas A&M University, College Station, Texas, USA

**Keywords:** Vector-borne bacteria, *Xylella fastidiosa*, Spiroplasmas, Phytoplasmas, Liberibacters, Prevention and control

## Abstract

Plant vascular tissues offer a unique habitat for piercing-sucking insects and the pathogens they carry. These insect-borne bacteria can infect thousands of plant species, threatening agriculture and food security. However, our understanding of their interactions with insect vectors and plant hosts is limited compared with that of the virus-vector-plant system, hindering the development of eco-friendly disease control methods. This review highlights recent findings on interactions between insects, plants, and key bacterial pathogens, including *Xylella fastidiosa*, Spiroplasmas, Phytoplasmas, and Liberibacters. We also discuss current management strategies and future directions.

## Introduction

Half of the global crop yield reduction is caused by direct insect feeding and insect-borne diseases [1, 2]. The plant vascular system, particularly the phloem, provides abundant carbohydrates, proteins, and amino acids for viral and bacterial microbes to colonize, although many of the hormones carried by the phloem are involved in systemic defense processes [3]. The primary role of the xylem is to transport water and mineral nutrients, which are absorbed by the roots from the soil, through the apoplast (the extracellular space situated between the plant cell plasma membrane and the cell wall) to other parts of the plant [4]. Despite the low nutrient content of xylem, plant pathogens have also been identified, living largely surrounded by dead cell tissue. Hemipteran insects have specialized stylets, which allow them to penetrate the plant vascular system. This gives them the chance to ingest sap with microbes and be carriers of pathogens; meanwhile, the pathogens require the assistance of insects to directly enter the vascular system. Whiteflies, aphids, and planthoppers are known to carry viral microorganisms, while psyllids transmit mainly bacterial pathogens. Pathogenic bacteria include *Xylella fastidiosa*, *Spiroplasma* spp., *Candidatus* Phytoplasma spp., and *Ca.* Liberibacter spp. [5]. Insect-borne bacterial diseases are among the major threats to high and stable yields in agriculture worldwide. Typical examples include citrus huanglongbing (HLB), which is caused by the citrus psyllid-borne *Liberibacter*. Since its discovery in 2005, Citrus HLB has drastically reduced citrus yields by 74% in Florida, the largest orange-producing state in the U.S. [6, 7]. In Italy, *X. fastidiosa* causes olive quick decline syndrome, resulting in widespread drying of olive trees and enormous economic losses [8].

**Fig. 1:**
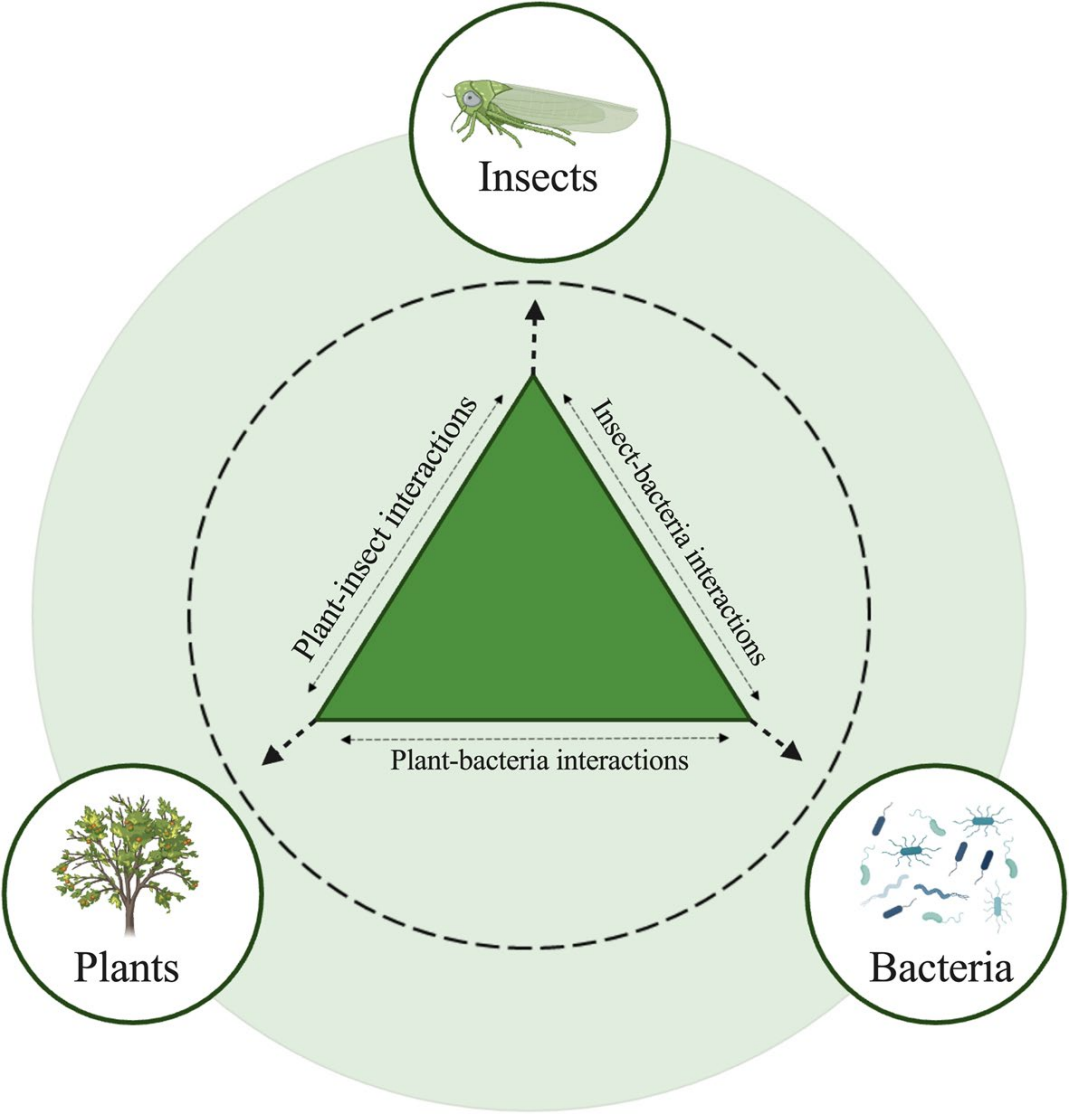
Three-way interactions among insect vectors, pathogens, and plants. In the intricate three-way interactions among insect vectors, pathogens, and plants, each component influences the others in a complex manner. Insect vectors, such as aphids and psyllids, are essential for transmitting plant pathogens, which in turn can alter plant characteristics to attract or repel these vectors. Additionally, pathogens may enhance vector performance by providing nutritional benefits or altering plant defense mechanisms, thus facilitating pathogen spread

Understanding the three-way interactions among vectors, pathogens, and plant hosts could provide novel strategies for effectively controlling the spread of pathogens. Plant-pathogen-insect interactions (Fig. 1) are complex processes in which pathogens can interact with insects and thus colonize their bodies, and transmission is achieved by pathogen-carrying insects that feed on plants. After delivery by insects, pathogenic bacteria can also interact with plants and colonize them through a range of infestation mechanisms, including the secretion of effector proteins. While the interactions between plant-pathogenic viruses and their hemipteran vectors have been extensively investigated, the relationships between plant-infecting bacteria and their hemipteran vectors remain relatively underexplored. Furthermore, few studies have been conducted on the identification and mechanisms of effector proteins produced by pathogenic bacteria due to technical limitations. For example, some bacteria are difficult to culture in vitro because of their need for fastidious growth conditions. [9].

In this review, we first introduce vector-borne bacteria and their host range. Second, we summarize the recent research progress on pathogenic mechanisms in plants, including pathogen-plant interactions, pathogen colonization, and pathogen immune evasion strategies. Third, we discuss the interactions between pathogenic bacteria and their insect vectors and review the limited information on the mechanisms underlying insect transmission of bacteria. Finally, techniques for preventing plant diseases and future directions are proposed.

### Vector-borne bacterial plant pathogens and their host range

**Fig. 2:**
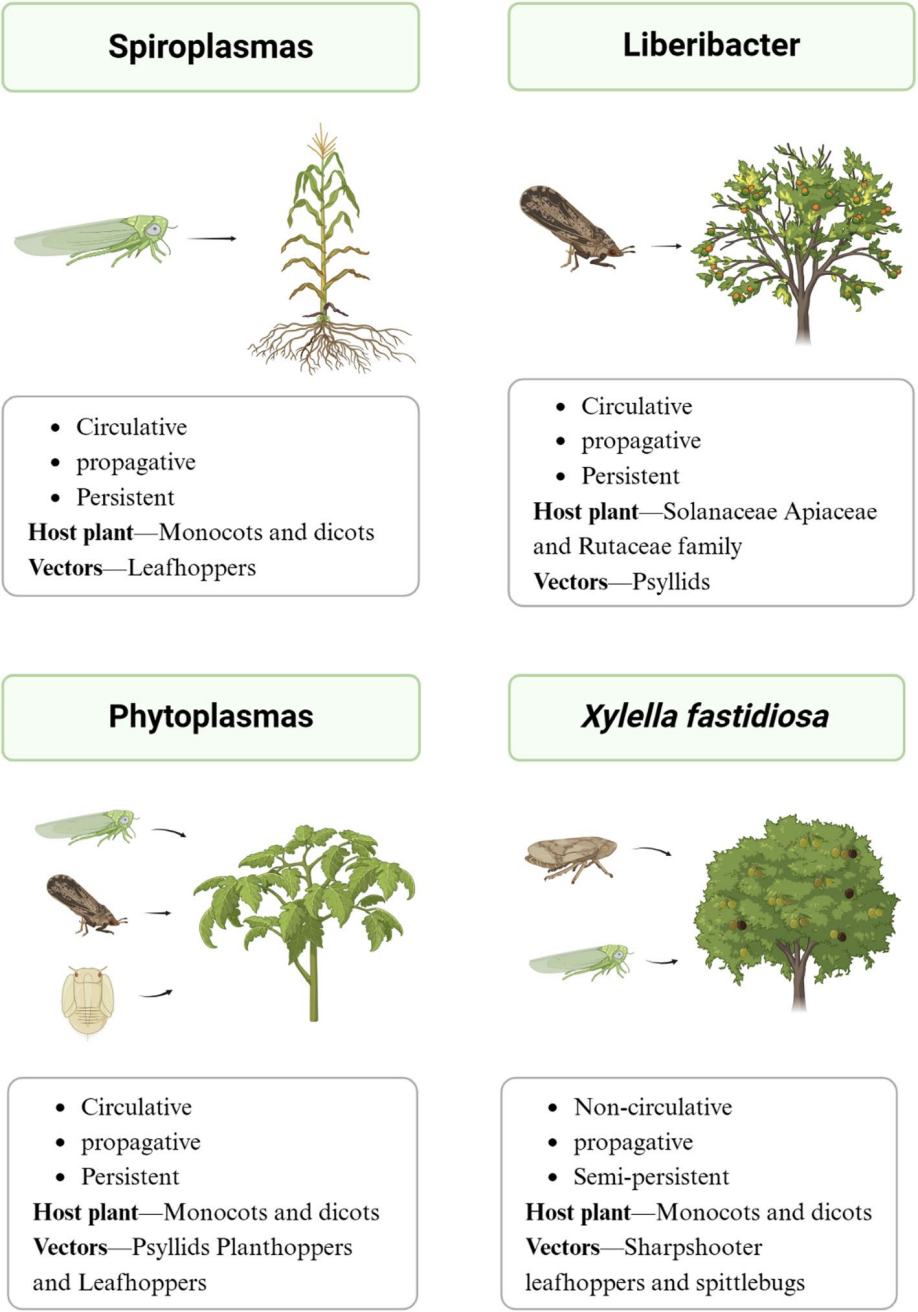
The range of vector-borne bacterial plant pathogens and their insect and plant hosts. Bacterial plant pathogens are transmitted primarily by leafhoppers, psyllids, froghoppers, and planthoppers and threaten thousands of crops and plants*. X. fastidiosa* has a semi-persistent and non-circulative association with its vectors. All known phloem-limited bacteria (e.g., Spiroplasmas, Phytoplasmas, and Liberibacters) appear to establish persistent and circulative associations with their respective vectors

Bacterial plant pathogens threaten numerous crops and plants, including the Solanaceae, Apiaceae, and Rutaceae families. Only a few certain groups of bacterial plant pathogens are transmitted by insects, most commonly of the suborder Auchenorrhyncha, including leafhoppers, froghoppers, and planthoppers. The psyllids from the suborder Sternorrhyncha are also critical vectors. Interestingly, the suborder Sternorrhyncha contains psyllids, aphids, whiteflies, and mealybugs, but only psyllids have been reported to be bacterial vectors, while the others transmit mainly plant viruses [10] (Fig. 2).

### Xylem-limited vector-borne bacteria

#### *Xylella fastidiosa*

*X. fastidiosa* (*Xf*) is a xylem-restricted Gram-negative gamma-ascorbic bacterium in the family *Xanthomonadaceae* that is phylogenetically most closely related to *Xanthomonas albilineans* [11]. *Xf* can be divided into at least five subspecies: typical examples include *Xf* subspecies *fastidiosa* (*Xff*), which causes Pierce's disease (PD) in grapes; *Xf* subspecies *pauca (*Xfp), which causes citrus chlorosis and olive quick decline syndrome; and *Xf* subspecies *sandyi* (*Xfs*). Recent studies have revealed that *X. taiwanensis* (*Xt*), which is most similar to *Xfs* and is thought to be a subspecies of *Xf*, is a new species [12, 13]. *X. fastidiosa*, whose geographic range was once thought to be limited to the Americas, was first detected in Italy in 2013 [14]. The disease led to the death of millions of olive trees in southern Puglia, and it was later discovered that the outbreak was caused by a Central American introduction of the pathogen in 2008 [15]. *X. fastidiosa* was subsequently found in several countries in Europe and was in fact introduced to Europe as early as 1993 [16].

*X. fastidiosa* is carried mainly by leafhoppers and spittlebugs and has been shown to infect more than 700 species of plants [17], including grape (Pierce’s disease), citrus (citrus variegated chlorosis), olive (leaf scorch), and almond (leaf scorch) plants. During insect feeding, *X. fastidiosa* enters the xylem from the feeding site and is transported to xylem tissues via Type IV pili-mediated twitching motility to obtain nutrient factors, including water, minerals, amino acids, alcohols, and sugars, for growth and reproduction [18, 19]. *X. fastidiosa*-infected plant hosts show symptoms of leaf tip drying, twig wilting, and even plant death with several pathogen infections [20]. *X. fastidiosa* can be cultured in vitro, and the conditions for *X. fastidiosa* growth can be met via a simply defined solid medium containing citrate, succinate, three amino acids (L-glutamine, L-asparagine, and L-cysteine), ammonia chloride, potato starch, GelRite, and mineral salts [21]. Although in vitro culture techniques have been optimized for media over a long period of time, the efficiency of *X. fastidiosa* growth remains low*,* perhaps because of metabolic inefficiency caused by the lack of key enzymes or virulence factors [22].

### Phloem-limited vector-borne bacteria

The phloem is a specialized vascular tissue composed of cell types such as sieve elements, companion cells, and phloem parenchyma cells that facilitate the transport of nutrients, primarily sugars and other organic molecules, throughout the plant. Owing to the rich nutrient composition of the phloem, many pathogenic bacteria colonize the region of plant phloem. The majority of phytoplasmas and liberibacters are vector-borne phytopathogens, whereas only some species of spiroplasmas are phytopathogens [23].

#### Spiroplasmas

Spiroplasmas belong to the class *Mollicutes*, which are helical, motile, cell- wall-less bacteria that are phylogenetically considered Gram-positive bacteria [24]. Although spiroplasmas have a broad host range and are found in both insects (such as orders of Hymenoptera, Coleoptera, Diptera, Lepidoptera, and Hemiptera) and plant hosts (e.g., citrus and carrot), three phytopathogenic spiroplasmas are transmitted by leafhoppers: *Spiroplasma citri*, *S. kunkelii*, and *S. phoeniceum*. *S. citri* was first discovered in 1970 and was the first vector-borne bacterium to be cultured [25, 26]. *S. citri* causes citrus stubborn disease, horseradish brittle root, and carrot purple leaf disease; *S. kunkelli* leads to maize stunt; and *S. phoeniceum*, which was isolated from periwinkle, presents typical symptoms of Mycoplasma-like organisms [27].

#### Phytoplasmas

In 1967, Doi et al. identified small, pleomorphic bacteria without cell walls in infected phloem tissues, which were subsequently named mycoplasma-like organisms (MLOs) [28] and were reclassified to phytoplasmas at the 9 th International Organization of Mollicutes (IOM) conference [29]. All phytoplasmas are now classified as *Candidatus* Phytoplasma species [30]. Unlike Spiroplasmas, which can be cultured in vitro, phytoplasmas are non-culturable bacteria. Thus, DNA samples are usually extracted from infected plants for phytoplasma genome sequencing, and the 16S rRNA gene sequences of phytoplasmas are mainly used for the molecular characterization of pathogens [31, 32].

Phytoplasmas, as emerging insect-borne bacteria, have received increasing attention in recent years. They can be transmitted by leafhoppers, planthoppers, and psyllids to infect thousands of plant species worldwide [33]. Plant diseases caused by Phytophthora usually include witches'broom, yellowing, dwarfing, wrinkling, stunted development or even death [34–36]. Interestingly, phytoplasmas often cause dramatic developmental changes in the plant host, including symptoms such as turning flowers into leaves (phyllody) or causing plants to develop dense and compact clusters of twigs and foliage (witches'broom) [37]. These diseases prevent plants from growing and reproducing normally and thus become a breeding ground for phytoplasmas and insect vectors.

#### Liberibacters

*Ca.* Liberibacter spp. is a phloem-limited Gram-negative bacterium belonging to the Rhizobiaceae family of α-Proteobacteria [38]. *Ca.* Liberibacter spp. has six subspecies: *Ca.* Liberibacter africanus (*C*Laf), *Ca.* Liberibacter americanus (*C*lam), *Ca.* Liberibacter asiaticus (*C*Las), *Ca.* Liberibacter solanacearum (*C*Lso), *Ca.* Liberibacte europaeus (*C*Leu), and *Liberibacter crescens* (*Lcr*). Studies have shown that they share a common ancestor and evolve into the non-pathogenic Lcr, which then evolves into the pathogenic *Ca.* Liberibacter [39].

Among them, the devastating citrus disease HLB is associated with three pathogens, *C*Laf, *C*lam, and *C*Las. The HLB agent is transmitted primarily by the Asian citrus psyllid *Diaphorina citri*. HLB was first reported in China in 1919, but the pathogen was not detected by electron microscopy until 1970 [6]. Infected citrus plants typically exhibit thinner crowns, yellow shoots, and mottled leaf spots [40]. HLB is present in several countries in Asia, sub-Saharan Africa, Indian Ocean islands, and the Americas and has recently been reported to occur in southern Europe in Portugal and Spain [40, 41].

*C*Lso contains at least six haplotypes: haplotypes A-E and U. Haplotypes A and B are transmitted mainly by the potato/tomato psyllid *Bactericera cockerelli* and cause diseases of Solanaceae, such as ‘zebra chip’ disease of potato. The above-ground symptoms are similar to those of ‘psyllid yellows’ and include stem twisting, node swelling, aerial tubers, vascular discolouration, and leaf scorching. Below-ground symptoms include collapsed stolons and enlarged lentil flaps on the tubers, and the medullary ray tissue becomes heavily streaked and becomes more pronounced in the tubers after deep-frying, giving rise to the characteristic name ‘zebra chip’ [42]. Haplotypes C, D and E are associated mainly with diseases of umbelliferous plants [43, 44], which are transmitted by the carrot psyllid *B. trigonica*. Haplotype U is found mainly in *Urtica dioica*. *C*Leu does not usually cause plant disease symptoms and is therefore not considered a plant pathogen [45].

Similarly, *Lcr* has not been reported to have pathogenic activity. *Ca.* Liberibacter spp. are fastidious and unculturable groups of bacteria, with the exception of *Lcr*, which may be caused by differences in the genome, with *Lcr* possessing more genes encoding thiamine and essential amino acids [46]. Therefore, transferring certain key genes to other *Liberibacter* species may be a way to make them culturable.

## Interactions between pathogenic bacteria and plant hosts

### Microbial modulation of plant metabolism

The phloem transport system provides a nutrient-rich environment that is rich in carbohydrates, proteins, and amino acids, which supports the survival of phloem-limited vector-borne bacteria. These phloem-restricted bacteria generally possess highly reduced genomes, lack essential metabolic pathways, and rely on their plant hosts for nutrients [47]. In contrast, xylem vessels primarily function in water transport and contain lower nutrient levels than the phloem does. Nevertheless, xylem-limited vector-borne bacteria have also been detected, despite the nutrient-poor conditions of the xylem.

*X. fastidiosa* causes up-regulation of molecular pathways associated with xylose formation and starch utilization in the plant. Excess xylose also causes water stress, limiting the water supply to the leaves and stomatal closures and reducing the photosynthesis of diffused incoming carbon dioxide. Thus, water and nutrient deficiencies in plants may lead to the eventual death of infected plants [48]. In phytoplasmas, starch catabolism is blocked in infected plants, leading to degradation of damaged chloroplasts, premature senescence of leaves, and reduced synthesis of gibberellins (GAs). The carbohydrate accumulation and sugar metabolism pathways have also been shown to be altered in infected plants [36, 49]. In *Liberibacter*-infected plants, metabolic pathways are also influenced. For example, in the early stages of HLB infection in citrus, active starch catabolism occurs in roots, and fatty acid profiles are altered in roots and leaves, with a more pronounced decrease in fatty acid content in roots [50].

### Pathogen movement and colonization in plants

*X*. *fastidiosa* produces plant cell wall-degrading enzymes, including a polygalacturonase and several endoglucanases, to degrade the xylem pit membrane, which connects adjacent xylem vessels to achieve systemic colonization of the xylem [51]. During this process, *X. fastidiosa* also produces virulence factors such as extracellular polysaccharides and adhesins, which promote biofilm formation to complete colonization. The cells of *X. fastidiosa* aggregate to create a'sticky'biofilm that firmly adheres to the xylem tubes and vascular system, enhancing the ability of the pathogen to establish and maintain colonization [52].

Phytoplasmas lack motility genes, and their phytoplasma membrane protein, immunodominant membrane protein (IMP), can bind to plant actin [53] and possibly facilitate the transport of phytoplasmas within the plant host [54, 55].

While in motion, Spiroplasmas cells display twitching movements, flexing, and rotation along the helical axis of the cell. Spiroplasmas swim via the formation and propagation of kinks. The spiroplasma has a tapered end structure that is thought to play an important role in the host attachment process [56]. For Liberibacters, previous studies have shown that *C*Las movement in the host plant phloem is not flagellum-mediated, but the bacteria can still be found in large numbers in sieve tubes after pathogen-carrying insects feed on plant stems [57], suggesting that bacteria might have other potential mechanisms of active movement called sliding movement [58].

### Pathogen-plant immune interactions

**Fig. 3:**
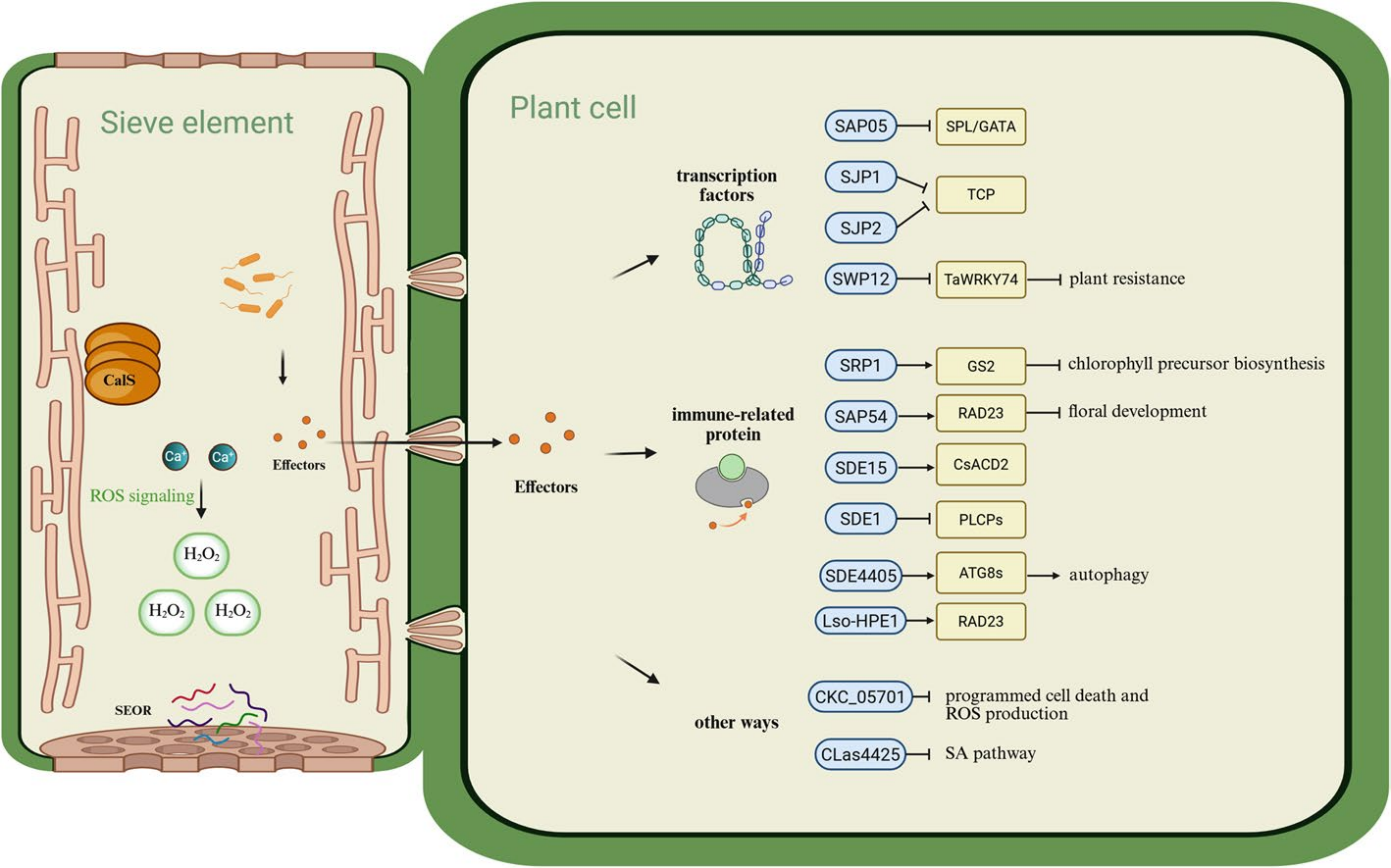
Plant immunity and immune evasion strategies of bacterial plant pathogens. Vector feeding and bacterial proliferation trigger the production of Ca^2+^, accumulation of Sieve Element Occluding Relatives (SEOR) proteins, and callose deposition through callose synthases (CalS). Bacterial pathogens secrete effectors to evade plant immune responses. For example, certain effectors from phytoplasmas (e.g., SAP05 and SAP11-like effectors SJP1 and SJP2) lead to the degradation of transcription factors. Additionally, effectors (e.g., SDE15 from *C*Las) can directly interact with immune-related proteins (e.g., CsACD2) to suppress plant immunity. Some effectors have also been shown to disrupt the salicylic acid (SA) signaling pathway in plants. The specific names and targets of these effectors are elaborated in the text

Plant immune defense usually involves the activation of surface-localized pattern recognition receptors (PRRs) by pathogen-associated molecular patterns (PAMPs) to induce PAMP-triggered immunity (PTIs), such as bacterial flagellin and fungal chitin [59]. Type III effectors suppress PAMP-triggered immunity (PTI) by interfering with the biogenesis of PRRs, the stability of PRRs, and the signalling components that lie downstream of PRRs. The suppression of PTI by effectors facilitates bacterial infection [60]. In addition, bacteria-secreted degrading enzymes may result in the production of damage-associated molecular patterns (DAMPs), which activate both plant immunity and the expression of PRRs [61]. Effectors can interfere with PTI, which results in effector-triggered susceptibility (ETS) [62]. Finally, plants have also evolved resistance proteins and triggered a third layer of defense,—called effector-triggered immunity (ETI),—where the ETI pathway induces a hypersensitive response through the production of reactive oxygen species (ROS), which cause cell death at the site of infection and thus limit the spread of the pathogen [63] (Fig. 3).

*X. fastidiosa*-infected grapevines produce tyloses in their bodies, which protrude into the xylem and are part of the plant's defense response, slowing or preventing pathogen movement within the xylem. However, overproduction of tyloses can lead to reduced hydraulic conductivity within the xylem, which is detrimental to the plant [64–66].

For phloem-inhabiting bacteria, flagellin from *C*Las and *C*Lso initiates PTI responses [67, 68], whereas phytoplasmas lack cell walls and flagella and therefore do not possess typical cell wall- and flagellum-derived PAMPs. Instead, phytoplasmas may elicit PTI-like reactions through internal PAMPs [69, 70]. Moreover, citrus huanglongbing is a pathogen-triggered immune disease in which *C*Las induces systemic and chronic immune responses in phloem tissue, including callose deposition and the production of ROS. Antioxidants and gibberellin can mitigate HLB symptoms [71]. Plants infected with *C*Lso, including lysM domain receptor-like kinase 4, exhibit defense mechanisms or stress responsiveness. This receptor kinase, featuring a lysine motif, acts as a cell surface receptor in chitin elicitor signalling pathways, thereby mediating innate immunity against specific fungal and bacterial pathogens [72]. However, the defense responses in tomatoes are inadequate to mitigate the disease symptoms and mortality associated with *C*LsoB infection, whereas they are sufficient to counteract the effects of *C*LsoA infection.

### Immune evasion of bacterial pathogens

Suppression of plant immunity is a common virulence strategy of pathogens. Pathogen-secreted effectors are major players in countering plant immune responses, and some pathogens can do so by secreting effectors directly into the host cytoplasm in the extraplasmic body space [73]. The interaction of cell-surface receptor ectodomains with plant PRRs is associated with the induction of immune responses and programmed cell death (PCD) [74]. Consistent with this view, the extracellular expression of cell wall-degrading enzymes and serine proteases triggered cell death in tobacco species, suggesting that PRRs are recognized in response to extracellular effector molecules [75]. However, current studies have shown that bacteria can take up metal ions through the T6SS to resist damage caused by oxidative stress. Furthermore, the T6SS is involved in bacterial adaptation to temperature and pH changes [76].

Bacteria normally secrete immunosuppressive effectors through the type III secretion system (T3SS), and type III secreted effectors are crucial in the disease process, as they act to disrupt vital host surveillance and defense mechanisms [77]. The *X. fastidiosa* genome lacks the T3SS, but studies have emphasized the critical role of the type II secretion system (T2SS) of *X. fastidiosa*, which secretes degradative enzymes that help maintain the infectious lifestyle of *X. fastidiosa* [78]. Spiroplasma has an incomplete Sec secretion pathway because the Spiroplasma genome lacks the gene encoding the SecB protein [79]. For phytoplasmas, a newly identified effector protein, SAP05, by Huang et al. was able to bind to the RPN10 subunit of the 26S proteasome of host cells, leading to degradation of the SPL/GATA transcription factor (Fig. 3), and the plant presented more lateral shoots, secondary meristems, and sterile flowers, which was more conducive to pathogen spread and infection [80]. In addition, the phytoplasma effector SAP11 [81] and two SAP11-like effectors, SJP1 and SJP2, promote lateral bud growth through the degradation of TCP transcription factors (Fig. 3) [82, 83]. An AY-WB effector protein, SAP54, induces alterations in floral development, leading to the emergence of leaf-like flowers [84]. Another effector, SWP12, which is secreted by phytoplasmas, also weakens plant resistance and promotes the colonization of *Ca. P. tritici* through degrading the transcription factor TaWRKY74 [85]. A recently reported effector protein 1 (SRP1) from rice orange leaf phytoplasma (ROLP) binds to the glutamine synthetase GS2, impairing chlorophyll precursor biosynthesis (Fig. 3). This process induces leaf yellowing in rice, attracting leafhopper vectors to increase pathogen transmission [86].

Similarly, *Liberibacter* secretes effector proteins to promote survival. Many bacteria deliver effector proteins to their hosts via the type III secretion system, and although *C*Las and *C*Lso have incomplete type III and type IV systems, they have the type I secretion system (T1SS) and all the essential components of the Sec mechanism [47]. Recent studies have revealed the ability of *C*Las to suppress plant immunity via the secreted protein SDE15, an effector containing a typical Sec-dependent secreted signal peptide that promotes *C*Las proliferation by targeting the citrus CsACD2 protein (a regulator of programmed cell death) (Fig. 3) [87]; mSECP8 (the mature form of SECP8) was hypothesized to interact with the *C*Las inducer of SDE1 as a key protein to manipulate plant immune responses [88]; SDE1 suppresses the activity of citrus papain-like cysteine proteases (PLCPs), thereby facilitating bacterial infection in plants [89]; and a *C*Las prophage-encoded effector targets ASCORBATE PEROXIDASE6 in citrus to facilitate bacterial infection [90]. The *C*Las4425 effector was shown to impair the salicylic acid (SA) signaling pathway in plants [91]. Autophagy is also a plant response to pathogen infection, yet pathogens have evolved multiple mechanisms to regulate autophagy to evade elimination. *C*Las is able to specifically degrade ATG8 family proteins via SDE3 in a GAPC1-dependent manner, thereby disrupting autophagy-mediated immunity (Fig. 3) [92]. In addition, the effector SDE4405 (CLIBASIA_04405) of *C*Las manipulates autophagy to promote bacterial infection. SDE4405 interacts with the ATG8- family of proteins (ATG8 s), and their interactions activate autophagy (Fig. 3) [93]. Furthermore, on the basis of a protein interactome, researchers succeeded in identifying 40 central nodal proteins of *C*Las that are involved in cell morphogenesis, may help *C*Las resist oxidative stress and are critical for *C*Las survival in the phloem [94].

In contrast to *C*Las, *C*Lso effectors have not been studied in depth. *Liberibacter* secretes proteins via the outer membrane vesicle (OMV) pathway and the SEC pathway. A recent study analyzing the expression of the *C*Lso effector pool revealed that the core effectors of *C*Lso are expressed mainly in the later stages of infection and that only a few effectors are required to suppress plant immune defenses [95]. A *C*Lso effector, hypothetical protein effector 1 (Lso-HPE1), from *C*Lso haplotypes A and B was able to inhibit the induction of cell death in plants [96]. Later, the same group reported that the virulence protein Lso-HPE1 in *C*Lso pathogenesis may disrupt the ubiquitin–proteasome system through direct interaction with the ubiquitin-like structural domain of the tomato RADIATION SENSITIVE23 (RAD23) protein (Fig. 3) [97]. Another *C*Lso effector, CKC_05701, was able to efficiently suppress programmed cell death and reactive oxygen species production [98].

## Interactions between pathogenic bacteria and insect vectors

**Fig. 4:**
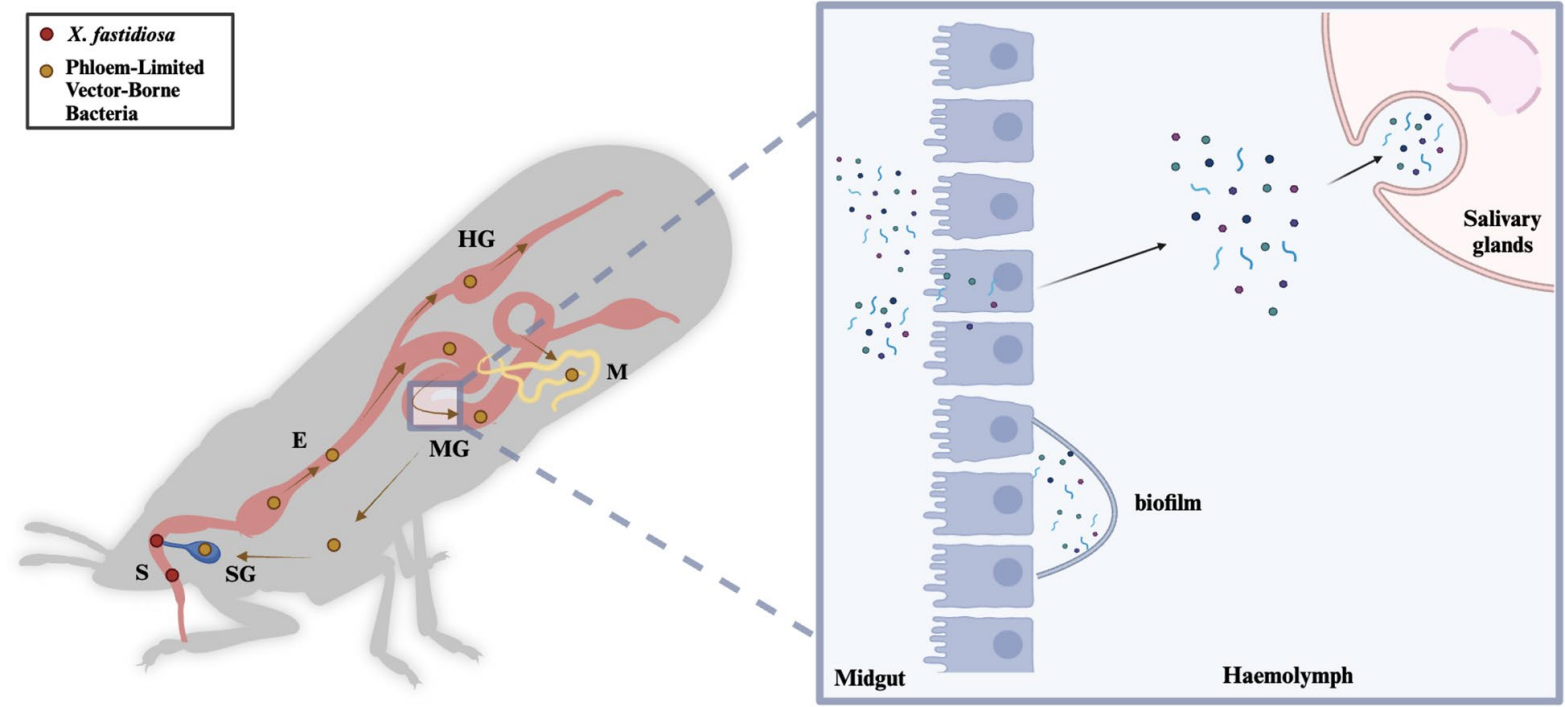
The transmission routes of vector-borne bacterial pathogens in the vector. *X. fastidiosa* (red) is not cyclic in insects and forms biofilms in the foregut region of insects after being obtained from plants and attached to them. Phloem-limited bacteria (yellow) pass through the intestinal barrier into the hemolymph and form biofilms on the intestinal surface. Free bacterial cells reach the salivary glands for the next transmission cycle

Among the described bacterial pathogens, *X. fastidiosa*, which is the sole recognized vector-borne xylem specialist, has a semi-persistent and non-circulative relationship with its vectors. This type of bacteria is likely to be more readily acquired and disseminated by vectors to a variety of host species. In contrast, all known phloem-limited bacteria (e.g., *Spiroplasma*, *Candidatus* Phytoplasma spp., *Candidatus* Liberibacter spp.) appear to establish persistent and circulative associations with their respective vectors (Fig. 4). Circulative pathogens cross the intestinal epithelium, colonize the insect hemolymph, and enter the salivary glands, where they are released into the plant with saliva during feeding by the vector insect. Insect vectors serve as alternative hosts for vector-borne bacteria, which are considered propagative (extracellularly or intracellularly) (Fig. 2).

The first stage of infestation in the interaction between pathogens and their insect vectors is marked by the pathogen's outer membrane making initial contact with the external surface of host cells. Thus, pathogenic bacteria have evolved a variety of surface adhesion mechanisms, which are indispensable for the formation of biofilms and for bacterial attachment and colonization within the host. Pathogen outer membrane components, mainly lipopolysaccharide (LPS), mediate the interaction between a pathogen and its environment. Bacterial attachment to host insects is dependent mainly on organelle action and the secretion of adhesion proteins. There are two main mechanisms by which bacterial pathogens further invade host cells: zipper and trigger [99]. The zipper mechanism uses bacterial surface proteins to bind to membrane-embedded receptors in the host cell, triggering a signaling cascade that reorganizes the actin cytoskeleton and ultimately leads to bacterial internalization, including lattice protein-mediated endocytosis [100]. The trigger mechanism employs a bacterial secretion system to deliver proteins to the host membrane to interact directly with cellular components that regulate actin dynamics.

The host immune system responds to microbial infections in a variety of ways, and the host immune response to pathogen invasion can be divided into humoral and cellular defense responses. The humoral defense response involves the synthesis of antimicrobial peptides and signaling pathways that regulate enzymatic cascades to influence hemolymph coagulation or melanization, whereas the cellular defense response involves hematopoietic cell-mediated immune responses, such as phagocytosis, colonization, and encapsulation [101–103]. The adaptation of vector insects to pathogens is usually assessed on the basis of their effects on vector survival and fecundity. Therefore, understanding microbial interactions in insect vectors is critical for disease control. In particular, strategies to disrupt pathogen transmission within vectors could be exploited.

### *X. fastidiosa* and insect vectors

The host insects of *X. fastidiosa* are mainly sharpshooters, leafhoppers, and spittlebugs. Recently, it was found for the first time that *Philaenus spumarius* (Hemiptera, Aphrophoridae) can serve as a vector insect for *X. fastidiosa* [104]. The pathogen is persistent and reproductive but not circulative in insects and forms biofilms in the foregut region of insects after being acquired from plants and attached to them. *X*. *fastidiosa* is unique in that it colonizes host insects; for example, *X. fastidiosa* degrades chitin, which is important for its ability to colonize insects [105]. The adhesion proteins of *X. fastidiosa* have been classified as fibrillar adhesins and afimbrial adhesins [106], which are key proteins for their colonization in the foregut of the vector, and in the case of *X. fastidiosa* subspecies *pauca* strains, adhesins such as XadA1 and XadA2 play important roles, with strong evidence that XadA2 has a high affinity for binding to host polysaccharides (chitin and cellulose) [107].

### Spiroplasmas and insect vectors

The spiroplasmas are transmitted by leafhoppers, and the vectors of *S. citri* are beet leafhoppers, *Neoaliturus tenellus* (Baker) in North America [108], and *Circulifer haematoceps* in the Mediterranean (Mulsant et Rey) [109]. The vector of *S. kunkelli* is *Dalbulus maidis* (DeLong) [110], and the vector of *S. phoeniceum* is *Macrosteles fascifrons* (Stål) [27]. The receptors in the gut lumen of insects mediate the occurrence of spiroplasmas endocytosis and interaction with cell membrane proteins [111]. The interaction between phosphoglycerate kinase (PGK) and actin promotes the colonization and delivery of *Spiroplasma* in insect cells [112]. Strong evidence is that *S. citri* internalized into insect cells is able to undergo morphological changes in the phloem sap and insect hemolymph by transforming from a spiral to a rounded form [113]. Spiroplasmas are generally believed to have a high degree of helicity. The adhesion proteins of spiroplasmas play a very important role in pathogen invasion. Common adhesion proteins such as spiralin, P58, P32, P89 (SARP1) and ScARPs (*S. citri* adhesion-related proteins) have been identified. The ScARPs and P89 (SARP1) proteins are associated with receptor-mediated endocytosis [114]. However, less attention has been given to spiculations, and knowledge of virulence proteins secreted by pathogens and their functions is still lacking. Several spiroplasma-associated adhesion proteins have been identified that we described earlier, and it was previously shown that the surface protein P86 of the spirochete *S. citri* acts as an adhesion protein during infection of the host *N. tenellus* [115].

### Phytoplasmas and insect vectors

Phytoplasmas are spread predominantly by phloem-feeding insects, including leafhoppers, planthoppers and psyllids [116, 117]. Recently, the mechanism by which phytoplasmas enter insect cells was shown to involve clathrin-mediated endocytosis [118]. Phytoplasmas interact directly with the host by secreting membrane proteins since they have no cell wall. The membrane proteins of phytoplasmas are classified into three types: immunodominant (Imp), immunodominant A (IdpA), and antigenic membrane proteins (Amps) [119], which occur during the intracellular transport of phytoplasmas, independent of insect adhesion processes [118]. Amps are representative of phytoplasma membrane proteins and are detected mainly on the surface of phytoplasma cells, where they form Amp-microfilament complexes with insect microfilaments and determine the ability of insects to translocate phytoplasmas [120, 121]. However, there are fewer studies in which phytoplasmas directly affect their hosts, and effectors are usually utilized to modulate the state of the plant to alter changes in the insect. For instance, the effector SAP11 has been shown to dampen JA signaling and increase insect fecundity in *Arabidopsis* [81]. Another effector, SAP54, from *Ca.* P. asteris promotes the degradation of the MADS-box protein, which inhibits flowering and increases insect colonization [122]. The effector protein SRP1 from ROLP suppresses the insect melanization immune response and promotes ROLP propagation [123]. In addition, feeding on phytoplasma-infected plants can increase the longevity and fecundity of both vector and non-vector leafhoppers [124].

### *Liberibacter* bacteria and insect vectors

The *Liberibacter* bacterial protein adhesin recognizes many different elements on the host cell surface [125] and mediates binding to insect adhesion protein receptors, and this protein–protein interaction plays a major role in the process of pathogen adhesion and movement. The movement of the HLB pathogen *C*Las in the host plant phloem is not flagellum-mediated [57], but the expression of flagellar and type IV bacteriophage mechanism genes is significantly up-regulated in *C*Las cells isolated from Asian citrus psyllids [126]. Understanding the titer and distribution of bacteria in insects is also critical for elucidating potential mechanisms by which bacteria affect host physiological functions. Interestingly, *C*Las was found to be located along the actin cytoskeleton of gut cells in the Asian citrus psyllid [127], which is similar to the observation of *C*Lso in psyllids [128, 129], suggesting that *Liberibacter* may rely on vector actin for colonization or translocation. Vector-borne pathogens are usually dependent on the host for metabolic and nutritional requirements because they lack certain essential genes. It has been shown that 95% of *C*Las genes are active during *D. citri* intestinal colonization, and these genes are associated with bacterial energy metabolism and the repair of bacterial genetic material, among other processes [127, 130]. These genes are essential for the survival and reproduction of bacteria. In addition, apoptosis in the gut of adult Asian citrus psyllids has been documented upon infection with *C*Las, whereas no apoptosis has been observed in the gut of nymphs [131, 132]. This apoptotic response in adults is hypothesized to diminish their capacity to acquire and transmit *C*Las, contrasting with the higher efficiency observed in nymphs [133].

*C*Lso haplotypes *C*LsoA and *C*LsoB are mainly potato zebra chip disease agents, while *C*LsoB titers increased more rapidly than *C*LsoA titer do in the adult psyllid gut,and furthermore, *C*LsoB was transmitted significantly more efficiently in the adult psyllid [134]. This difference could be related to distinct immune responses. Indeed, potato psyllids mount distinct gut immune responses against these two haplotypes [135]. *C*LsoA represses the PI3 K-Akt pathway and activates the FoxO signaling pathway, whereas *C*LsoB up-regulates the mTOR and MAPK pathways. Furthermore, two inhibitors of apoptosis were up-regulated by *C*LsoB, suggesting that *C*LsoB might repress apoptosis in the psyllid gut. Indeed, no evidence of apoptosis has been reported in the gut of potato psyllid adults [136]. Tang et al. (2020) found *C*Lso inhibit the apoptotic response in the psyllid gut by triggering the expression of anti-apoptotic gene IAPP5.2 during the early stage of infection. This may increase *C*Lso acquisition in gut cells and facilitate CLso transmission by potato psyllids [137]. In addition to studies on immunity, several studies have revealed metabolic changes in host insects following infection. *C*Lso evades the host immune defense response by secreting glycerophospholipids in psyllids, which was confirmed by the recent finding of a significant increase in glycerophospholipid content in *C*Lso-infected psyllids [138]. However, there is also evidence that infection by *C*Lso can suppress the survival of insects, thereby affecting the spread of plant diseases [139].

### Bacterial pathogens and insect symbionts

Insects contain symbiotic microorganisms, which may interact with pathogenic bacteria and influence infection [140]. The Asian citrus psyllid *D. citri* has three main symbiotic bacteria in its body: *Ca.* Carsonella ruddii, *Ca.* Profftella, and *Wolbachia*. According to the gut metagenome of *D. citri*, *C*Las infection significantly affects the commensal bacterial community in the host [141]. *Wolbachia* in insects is positively correlated with the *C*Las titer [142], and *Wolbachia* can be detected in the salivary glands of *D. citri*, which affects the protein composition of saliva; these proteins may be involved in key functions in blocking pathogen transmission [143]. There are studies utilizing citrus endophytes to effectively control citrus HLB [144]; although the mechanism of pathogen inhibition by endophytes needs to be further explored, these studies have provided valuable insights into the biological control of citrus HLB. In another case, *Wolbachia* affects the acquisition and transmission of the zebra chip disease agent *C*Lso in potato/tomato psyllids, *B. cockerelli*. The psyllids lacking *Wolbachia* are less efficient at acquiring and transmitting pathogens [145].

### Vector-pathogen-plant three-way interactions

During insect feeding, plants are able to sense insect-derived salivary proteins to activate a series of signaling pathways. Insect salivary proteins are divided into elicitors and effectors, and the elicitors are also known as herbivore-associated molecular patterns (HAMPs) to induce the first layer of plant defense [146]. The main plant defenses triggered by insect elicitors include activation of the JA and SA pathways, ROS bursts, callose deposition, Ca^2+^ influx, and MAPK activation [147]. In addition, insects secrete effectors that are used to inhibit plant defense responses and induce ETI defense in plants.

Pathogens secrete effectors that can attract vector insects by manipulating host plant development and regulating plant volatiles, thereby facilitating their own spread [148]. However, little is known about the molecular mechanisms by which pathogens mediate changes in plants to attract vector insects. In recent years, research in this area has focused on phytoplasmas, but only a few of the effectors of phytoplasmas [149], including SAP11, SAP54, and SAP05, have a defined function [80, 150, 151]. Specifically, SAP11 interferes with the synthesis of plant defense hormones and enhances leafhopper fecundity, thereby facilitating phytoplasma transmission [81]. SAP54 degrades proteins during plant development to cause plants to exhibit sterility symptoms and become more attractive to leafhoppers [122], and SAP54 also interacts with the host plant MADS-box transcription factor SVP, a conserved host regulatory protein, to manipulate leafhopper vector behavior by increasing female attraction [152]. SRP1 effectors impair chloroplast synthesis-related enzymes, leading to leaf yellowing symptoms that attract more leafhoppers [153]. In addition, *C*Las induces citrus to release a specific plant volatile that indirectly attracts the vector *D. citri* [154]. *C*Las also influences the release of plant metabolites, volatile organic compounds (VOCs), to regulate the preference of vector insects for uninfected plants [155]. All these findings facilitate our understanding of the devastating issues caused by insect-pathogen-plant interactions.

## Progress in the control of bacterial vector-borne plant diseases and future directions

In terms of management and control measures for plant diseases caused by pathogens, the common strategy is chemical control based on insecticides and antimicrobials [156, 157]. In addition, antibiotic treatment is helpful for managing HLB- or phytoplasma-infected plants [158, 159], but different antibiotics are employed to target specific bacterial pathogens. For example, the use of antibiotics, including oxytetracycline and streptomycin, was approved by the U.S. Environmental Protection Agency (EPA) for the treatment of HLB in Florida because of significant economic losses throughout the citrus industry [160–162]. Trunk injection with antibiotics in citrus is more efficient than foliar spray, even after the use of adjuvants [162]. In addition, phytoplasmas can be eliminated from infected plants by tetracycline and rifampicin [54]. Clarithromycin also effectively suppresses the viability of apple proliferation phytoplasma in explant cultures [163]. Defense-triggering peptides, both synthetic and naturally sourced, are considered potential candidates for plant disease control, with the most studied synthetic peptide being flg22-OH, and recent studies have shown that the internal administration of flg22-NH2 triggers a stronger defense response in plants against *X. fastidiosa* [164]. *Streptococcus lactis* peptide is a naturally occurring antimicrobial peptide that is less expensive to produce than synthetic antimicrobial peptides and achieves potent and rapid antimicrobial activity by disrupting the *X. fastidiosa* lipid bilayer [165], which has previously been shown to be effective against drug-resistant bacteria [166]. In addition, Huang et al. characterized MaSAMP, a heat-stable antimicrobial peptide from HLB-resistant Microcitrus that both kills *C*Las and induces plant immunity [167]. However, bacterial pathogens can produce protective biofilms that are resistant to antimicrobial compounds and make it difficult for them to reach vascular tissues [168]. In addition, antibiotic treatment is helpful for managing HLB- or phytoplasma-infected plants [158, 159], but different antibiotics are employed to target specific bacterial pathogens. It is also critical to effectively eliminate infected plants once they are found.

Well-designed antimicrobial compounds, which are designed to inhibit key enzyme activities in prokaryotic pathogens, may be effective ways to stop the growth of pathogens. The exploration of new sources of resistance, whether derived from wild relatives or obtained through genetic engineering, may play a key role in protecting plants from pathogens and vectors [169–176]. In addition, the enhancement of plant resistance by CRISPR/Cas9 gene editing technology is underway to develop environmentally sustainable alternatives [177]**.** Moreover, enhancing plant resistance can be accomplished through the development of natural plant immunomodulators such as salicylic acid (SA), SA analogues, and brassinosteroids, which have been tested to control HLB by inducing plant immunity [178, 179]. Enhancing plant resilience by fine-tuning metabolism-based responses, maintaining phytohormone homeostasis, and modulating plant growth performance is also a promising alternative solution to improve plant tolerance to adverse conditions. Indeed, one recent study revealed that Gamma-Aminobutyric Acid (GABA) may be involved in phytohormone-based defense responses and other phytohormone-based defense responses against *C*Las and its insect vector [180]. Recent interest in light engineering and light-mediated plant resistance mechanisms will provide new directions for developing plant disease control strategies based on light treatments and crop breeding [181].

Controlling the spread of disease remains a major task because numerous pathogens cannot be cultured or genetically manipulated under laboratory conditions. Early identification is essential to stop the spread of disease. Recent studies have focused on the discovery of small molecules and peptides that can act as antimicrobial substances, and these studies offer hope for pathogen control. New research efforts are needed to explore the complex relationships among pathogens, insects, and plants. Understanding this relationship helps us understand how they have interacted with each other over the course of evolution, which is critical to the development of new technological tools to counteract the global devastation caused by pathogens and diseases carried by insects.

## Data Availability

Data sharing is not applicable to this article.
